# The 5^th^ Annual Heart in Diabetes Conference (part 2)

**DOI:** 10.1111/1753-0407.13251

**Published:** 2022-01-24

**Authors:** Zachary Bloomgarden

**Affiliations:** ^1^ Department of Medicine, Division of Endocrinology, Diabetes, and Bone Disease Icahn School of Medicine at Mount Sinai New York NY USA

At the 5^th^ Annual Heart in Diabetes (HiD) Conference, held in New York from 10 to 12 September 2021, a variety of topics were addressed related to the cardiovascular (CV) risks and CV complications of diabetes. This is the second of a two‐part summary of some of the presentations at the meeting, reviewing approaches to therapy.

Michael Farkouh, Toronto, Ontario, Canada, discussed the interaction of optimal medical therapy and revascularization strategies in patients with diabetes and coronary artery disease (CAD). After percutaneous coronary intervention (PCI) for CAD, nontarget lesions are frequent sites of atherosclerotic cardiovascular disease (ASCVD) events,^1^ suggesting that stent placement alone may not be sufficient treatment for persons with CAD. Farkouh reviewed the Future Revascularization Evaluation in Patients with Diabetes Mellitus: Optimal Management of Multivessel Disease (FREEDOM) study, comparing stent placement with coronary artery bypass graft (CABG) surgery and showing over 5 years 18.7% of enrolled patients with multivessel disease had myocardial infarction, stroke, or mortality after CABG, while these outcomes occurred in 26.6% of those having PCI.^2^ He noted that this and other studies suggest the importance of optimized medical therapy of CAD^3^ and acknowledged that future studies will need to include sodium glucose cotransporter 2 inhibitors (SGLT2i) and glucagon‐like peptide 1 receptor agonists (GLP‐1RA), but pointed out that even in these clinical trials there is lack of uniformity in achieving diabetes, lipid, and blood pressure goals, so that a major clinical challenge is the delivery of appropriate treatment to the large number of persons with diabetes and CAD. Interestingly, his colleague Lucas Godoy, Toronto, reviewed a meta‐analysis performed with Farkouh of patients with diabetes undergoing PCI or CABG, followed for 4 years, showing similar outcome with low‐density lipoprotein (LDL) cholesterol <70 and 70 to <100 mg/dL, with both groups having 23% lower event rates than those with LDL cholesterol ≥100 mg/dL,^4^ although another study from the group showed that after PCI there is a gradient of adverse outcome from LDL cholesterol below 70 to LDL between 70 and <100 to LDL of 100 and over,^5^ supporting that the LDL cholesterol goal is below 70 mg/dL for diabetes with ASCVD.

Peter Grant, Leeds, UK, discussed antiplatelet and anticoagulant treatment approaches for persons with diabetes. In a study of more than 15 000 persons with diabetes not having evidence of ASCVD randomized to aspirin 100 mg daily or placebo, serious vascular events occurred in 1.1% fewer persons over a mean 7.4‐year follow‐up, but major bleeding (primarily gastrointestinal) occurred in 0.9% more,^6^ leading Grant to suggest that the choice of whether or not aspirin should be given in such a primary prevention setting should be personalized. In acute coronary syndrome, aspirin combined with one of the P2Y12 platelet adenosine diphosphate receptor inhibitors clopidogrel, prasugrel, or ticagrelor is recommended, with evidence that prasugrel leads in general to optimal outcome.^7^ The combination can be continued for up to 3 years unless there is high bleeding risk, after which P2Y12 inhibitors alone may lead to better outcome. At age over 75 years, or with prior stroke or transient ischemic attack, prasugrel increases cerebrovascular event rates, and clopidogrel or ticagrelor may be preferable. With peripheral arterial disease, a different approach may be preferred with low‐dose rivaroxaban and aspirin.^8^

Silvio Inzucchi, New Haven, CT, discussed stroke prevention in diabetes, pointing out the association of worse glycemic control with risk of fatal stroke in the United Kingdom Prospective Diabetes Trial,^9^ with metformin associated with lower risk of stroke than conventional treatment, and as well with lower risk of stroke than insulin or sulfonylurea treatment.^10^ Pioglitazone was associated with ~50% reduction in stroke risk among persons with diabetes and prior stroke in the Prospective Pioglitazone Clinical Trial in Macrovascular Events (PROactive)^11^ and with 25% reduction in the combined endpoint of myocardial infarction and stroke among persons with insulin resistance (but not diabetes) and prior stroke in the Insulin Resistance Intervention After Stroke (IRIS) randomized clinical trial.^12^ Among newer glucose‐lowering agents, GLP‐1RA are associated with 14% reduction in stroke rate in clinical trials of these agents.^13^ Inzucchi concluded that diabetes treatment guidelines might differ in persons with history of stroke rather than other forms of ASCVD, an important point in view of the relative infrequency of current use of pioglitazone despite its apparent benefit in this setting.

Pam Taub, San Diego, CA, discussed the question of optimal diets for prevention of cardiovascular disease (CVD). Evidence‐based recommendations include appropriate calorie restriction based on age, sex, and activity level, for dietary fiber, for the Mediterranean and DASH diet approaches, and, perhaps, for time‐restricted eating approaches.^14^, ^15^ She suggested that fasting can be considered a metabolic switch from metabolism of glucose to that of fatty acids and ketones,^16^ with evidence of ketogenesis being associated with improvement in cardiac energetics.^17^ Taub raised the interesting concept that a similar mechanism may underly the benefit of SGLT2 inhibitors,^18^ although with the potential that fasting may be problematic with the use of these agents. Taub turned to a different topic, that of the amine oxide trimethylamine N‐oxide (TMAO), produced by gut bacteria from choline, lecithin, and L‐carnitine‐rich foods such as fish, meat, eggs, and dairy products, associated with CVD and diabetes, with evidence that TMAO levels decrease with fasting,^19^ a potential mechanism benefit of intermittent fasting approaches.

Donna Ryan, New Orleans, LA, further addressed issues related to obesity, reviewing expected benefits, and current approaches to pharmacotherapy, noting the important difference between semaglutide, causing mean weight loss of 15% to 17%, and older medicines for appetite regulation such as phentermine/topiramate, naltrexone/bupropion, and liraglutide, all causing weight loss of 6% to 11%. Ryan noted the infrequent prescribing of anti‐obesity medications, only given to approximately 1% of eligible patients, in part because of financial barriers such as high co‐pays and the need for prior authorization for prescriptions.^20^ The Semaglutide Effects on Heart Disease and Stroke in Patients with Overweight or Obesity (SELECT) study is an ongoing randomized, double‐blind, parallel‐group trial of semaglutide 2.4 mg subcutaneously once weekly added to standard of care, which may show the beneficial effect of weight loss in reducing major adverse cardiovascular events in approximately 17 500 patients with established CVD and overweight or obesity but without diabetes.^21^, ^22^ Ryan also discussed the concept that weight normalization is not necessarily the goal, reviewing studies showing that substantial decrease in visceral fat, in intrahepatic fat, and in markers of glycemia, insulin sensitivity and action, liver function, dyslipidemia, and inflammation can be seen with weight loss to the degree found with semaglutide.^23^ Indeed, in the Diabetes Prevention Program, ~10% weight loss optimally reduced diabetes likelihood,^24^ and in the Diabetes Remission Clinical Trial (DiRECT) of 306 persons with type 2 diabetes randomized to intensive diet intervention, those with weight gain vs losing 0 to 5 kg, 5 to 10 kg, 10 to 15 kg, and >15 kg had no, 7%, 34%, 57%, and 86% rates of diabetes remission without need for medications.^25^ Furthermore, secondary analysis of the Look AHEAD randomized clinical trial of lifestyle intervention for persons with diabetes showed that those with ≥10% weight loss had ~20% reduction in CVD outcomes.^26^

Stephen Wiviott, Boston, MA, discussed the effect of SGLT2i on atrial fibrillation, reviewing the uncertainty as to the pathogenesis of this arrhythmia in diabetes, potentially involving alteration in the balance between parasympathetic and sympathetic tone due to autonomic neuropathy and insulin resistance. Certainly, the coexistence of diabetes with atrial fibrillation increases stroke risk. In analysis of a trial of more than 17 000 persons with diabetes, administration of dapagliflozin vs placebo was associated with 19% reduction in first event and a 33% reduction on subsequent events of atrial fibrillation.^27^ Similar reduction in atrial fibrillation has been reported with canagliflozin^28^ and in a meta‐analysis of more than 50 000 persons in clinical trials of SGLT2i.^29^ Wiviott noted a recent presentation suggesting reduction in ventricular arrhythmias with dapagliflozin and suggested that a variety of mechanisms may contribute to such antiarrhythmic effects of SGLT2i, including decongestive effect, blood pressure lowering, weight loss, A1c benefit, direct cardiac effects on remodeling, on sympathetic tone, on oxidative stress/inflammation, and on epicardial fat.

Rajiv Agarwal, Indianapolis, IN, reviewed an analysis of the Finerenone in Reducing Kidney Failure and Disease Progression in Diabetic Kidney Disease (FIDELIO‐DKD) study on the effect of this agent on CV outcomes in this study of 5674 persons with type 2 diabetes and diabetic kidney disease based either on having microalbuminuria with estimated glomerular filtration rate (eGFR) 25 to 59 or on having eGFR up to 74 with macroalbuminuria,^30^ a group clinically recognized to be at extremely high risk of CVD complications. The composite CV outcome of CV death, myocardial infarction, stroke, or hospitalization for heart failure occurred in 13% of those receiving finerenone vs 14.8% of those receiving placebo, a significant 14% reduction, suggesting an important potential role of the agent in reducing CV events. The CV outcome benefit of finerenone appears particularly related to reduction in heart failure,^31^ suggesting this, along with the SGLT2i, to be important additions to our therapeutic armamentarium.

Sam Dagago‐Jack, Memphis, TN, discussed an aspect of what he termed the “African American Paradox”: that Blacks in the United States have higher blood pressure and rates of diabetes, but lower levels of LDL and triglyceride and higher levels of high‐density lipoprotein cholesterol, yet their CVD mortality is higher than that in Whites. Part of the issue, he suggested, may be lesser degree of control of risk factors,^32^ but he noted that there also appears to be lower provision of acute and effective optimized CV treatment. Dagago‐Jack concluded by noting that the profile of SGLT2i “suggests that these agents may help reduce disparities in CKD, HF and CVD mortality,” but he pointed out the underrepresentation of Blacks and Hispanics in CV outcome trials, with such broader enrollment needed to realize the promise of these and other novel agents.
